# Supplementary material for: Splice-Junction-Based Mapping of Alternative Isoforms in the Human Proteome
Source: Cell Rep. Author manuscript; Available in PMC 2020 Jan 15. (PMC6961840; doi:10.1016/j.celrep.2019.11.026)
Supplement: 2 [file NIHMS1546469-supplement-2.zip › DF1/PXD006675/RightAtrium/RightAtrium_41_OTUD5_GALVPPPDADPANEPPPPGPMPPAPR.pdf]

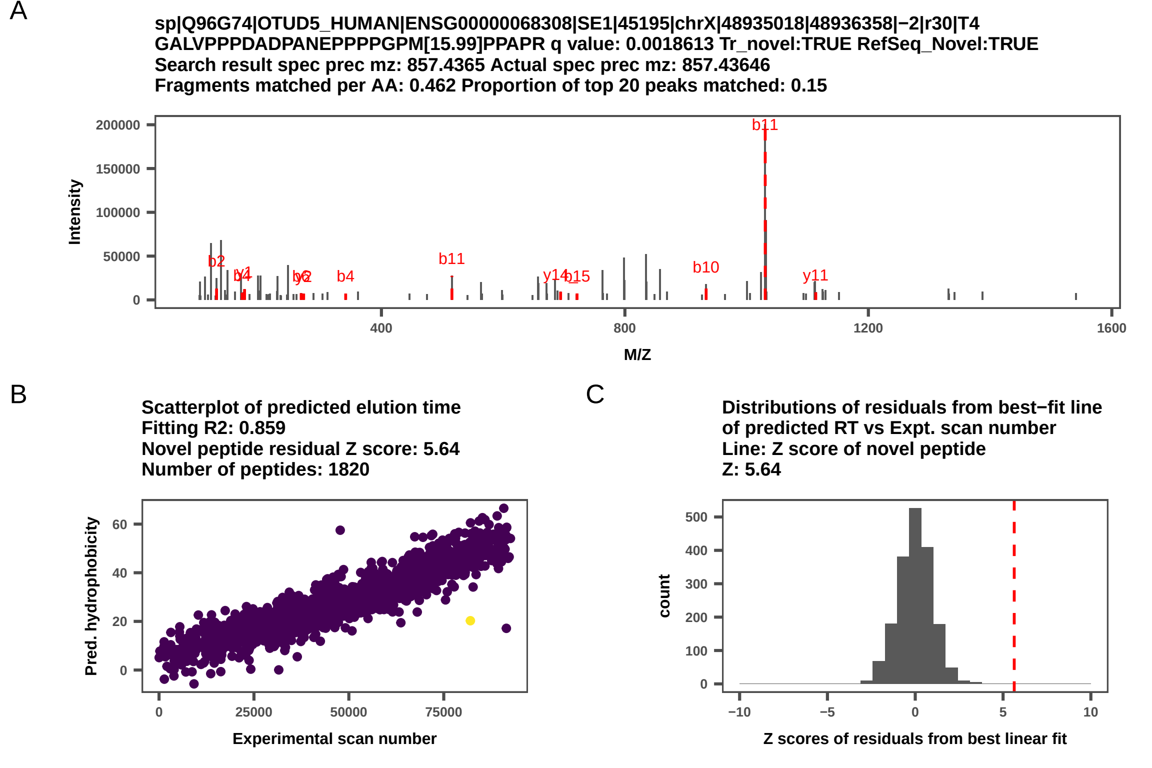
A
sp|Q96G74|OTUD5_HUMAN|ENSG00000068308|SE1|45195|chrX|48935018|48936358|−2|r30|T4
GALVPPPDADPANEPPPPGPM[15.99]PPAPR q value: 0.0018613 Tr_novel:TRUE RefSeq_Novel:TRUE
Search result spec prec mz: 857.4365 Actual spec prec mz: 857.43646
Fragments matched per AA: 0.462 Proportion of top 20 peaks matched: 0.15
Intensity
200000
150000
100000
50000
0
b11
b2
b4
y1
b6
y2
b4
b11
y14
b15
b10
y11
400
800
1200
1600
M/Z
B
Scatterplot of predicted elution time
Fitting R2: 0.859
Novel peptide residual Z score: 5.64
Number of peptides: 1820
Pred. hydrophobicity
60
40
20
0
0
25000
50000
75000
Experimental scan number
C
Distributions of residuals from best−fit line
of predicted RT vs Expt. scan number
Line: Z score of novel peptide
Z: 5.64
count
500
400
300
200
100
0
−10
−5
0
5
10
Z scores of residuals from best linear fit
